# Ferrous lactate-loaded hydrogels induce iron-dependent non-canonical ferroptosis in *Cutibacterium acnes*: a novel therapeutic strategy for acne vulgaris

**DOI:** 10.1016/j.mtbio.2026.103174

**Published:** 2026-04-29

**Authors:** Rui Wang, Haizhen Mo, Liangbin Hu, Jintae Lee, Lishan Yao, Shurui Peng, Min Zhang, Wei Zhou, Hongbo Li, Jiayi Zhang

**Affiliations:** a School of Food Science and Engineering, Shaanxi University of Science and Technology, Xi'an, China; b School of Chemical Engineering, Yeungnam University, Gyeongsan, Republic of Korea; c School of Food Science, Henan Institute of Science and Technology, Xinxiang, China

**Keywords:** FeLac-loaded hyaluronic acid hydrogels, *Cutibacterium acnes*, Ferrous lactate, Ferroptosis, Acne treatment

## Abstract

Acne vulgaris is a common inflammatory skin condition that affects up to 85% of adolescents and is primarily driven by *Cutibacterium acnes* (anaerobic bacteria, formerly *Propionibacterium acnes*). With the rise of antibiotic resistance, research on alternative antimicrobial agents that can address anaerobic bacteria resistance is becoming increasingly important, especially for patients who cannot use systemic antimicrobial therapy. In this study, a hyaluronic acid (HA)-based hydrogel incorporating ferrous lactate (FeLac) was developed as a non-antibiotic antimicrobial strategy targeting anaerobic pathogens to evaluate the bactericidal effects of FeLac on *C. acnes*. FeLac-loaded HA hydrogels exhibited excellent mechanical properties, good biocompatibility, superior storage stability, and robust antibacterial activity through sustained Fe^2+^ release, suggesting their potential as topical antimicrobial formulations. In a rat model of *C. acnes*-induced acneiform lesions, topical application of FeLac-loaded hydrogel significantly reduced inflammation and promoted lesion resolution through Fe^2+^ release, with efficacy comparable to conventional topical antibiotics. Mechanistically, FeLac (200 μM) treatment significantly increased intracellular iron levels, induced iron-dependent non-canonical ferroptosis, disrupted key pathways related to translation, biosynthesis, and cell wall integrity, and induced marked morphological alterations, achieving a bactericidal rate exceeding 99.9% after 3 h of treatment. These findings indicate that the FeLac-loaded hyaluronic acid (HA) hydrogel represents a promising non-antibiotic platform for treating acne and anaerobic bacterial infections via an iron-dependent ferroptosis-mediated pathway, demonstrating significant potential in dermatology and the cosmetics industry.

## Highlights

• FeLac-loaded HA hydrogel offers sustained, non-antibiotic acne treatment.
• Induces non-canonical, ROS-independent ferroptosis in anaerobic *C. acnes*.
• Achieves >99.9% bacterial killing through Fe^2+^-mediated mechanisms.
• Shows comparable efficacy to topical antibiotics in vivo.
• Supports skin healing with excellent biocompatibility and topical safety.

## Graphical abstract

**Figure:**
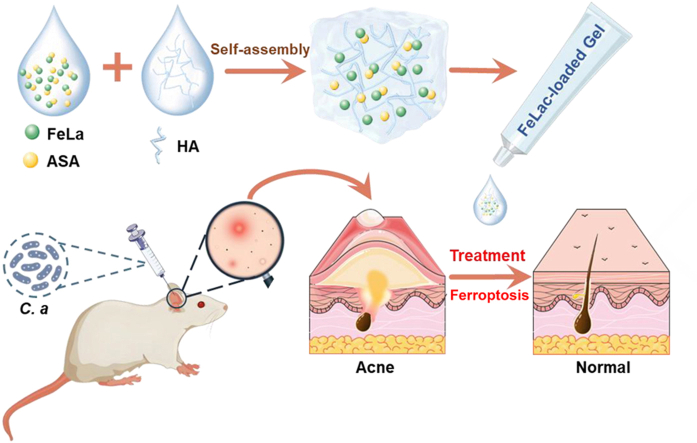

## 1 Introduction

Acne vulgaris is a prevalent chronic inflammatory skin condition, particularly common during adolescence, affecting approximately 9% of the global population and nearly 85% of teenagers [1,2]. Ranked as the eighth most burdensome global disease, severe acne can lead to permanent scarring and exert profound physical and psychosocial impacts, significantly increasing the risk of depression and suicide [3,4]. The pathogenesis of acne is multifactorial, involving sebaceous gland hyperactivity, abnormal follicular keratinization, dysbiosis within the pilosebaceous microbiota, and dysregulation of immune system [5]. Colonization by *Cutibacterium acnes* within the pilosebaceous follicle has long been recognized as a key contributor to acne development [6,7]. Due to its chronic nature, acne frequently requires long-term or maintenance therapy to reduce the likelihood of recurrence [8]. For decades, both systemic and topical antibiotics have been the mainstay of acne treatment by targeting *C. acnes* and mitigating inflammation [9,10]. However, this prolonged and recurrent use of antibiotics has inadvertently led to an alarming increase in antimicrobial resistance (AMR) [11,12]. Given the impending antibiotic resistance crisis, there is an urgent need to develop a non-antibiotic antibacterial hydrogel for the treatment of acne and other anaerobic bacteria infections.

Iron, an essential transition metal, plays a pivotal role in various cellular processes, including the maintenance of skin integrity, mucosal membranes, hair, and nails [13,14]. Excess labile iron can generate reactive oxygen species (ROS) through the Fenton reaction, triggering ferroptosis and oxidative stress-associated cell death in mammalian cells [15]. Intriguingly, ferroptosis-like mechanisms have also been identified in prokaryotes, where iron-dependent ROS accumulation induces bacterial cell death [16,17]. Inducing ferroptosis in bacteria not only offers an alternative to conventional antibiotics but also represents a promising strategy for overcoming AMR through a unique bactericidal mechanism [18,19]. Despite this potential, most existing studies have focused on inducing iron-dependent ferroptosis in aerobic pathogenic microorganisms through oxidative stress, while limited research on whether anaerobic microbes can also undergo iron-dependent ferroptosis as an alternative therapeutic strategy.

Hyaluronic acid (HA), an anionic glycosaminoglycan, is predominantly found in the extracellular matrix of various tissues, including connective, epithelial, and neural tissues [20]. HA-based hydrogels are increasingly studied as drug delivery systems due to their intrinsic biocompatibility, hydrophilicity, and tunable drug release profiles [21]. When complexed with cationic compounds, HA enhances the stability of the resulting structures [22]. Such interactions can improve the mechanical properties of hydrogels and other biomaterials, making them more resistant to degradation and better suited for biomedical applications [23]. Hence, HA as the skeleton of hydrogel to develop a versatile HA-based hydrogel system has potential significance, particularly for the treatment of acne and infections caused by anaerobic bacteria.

**Fig. 1:**
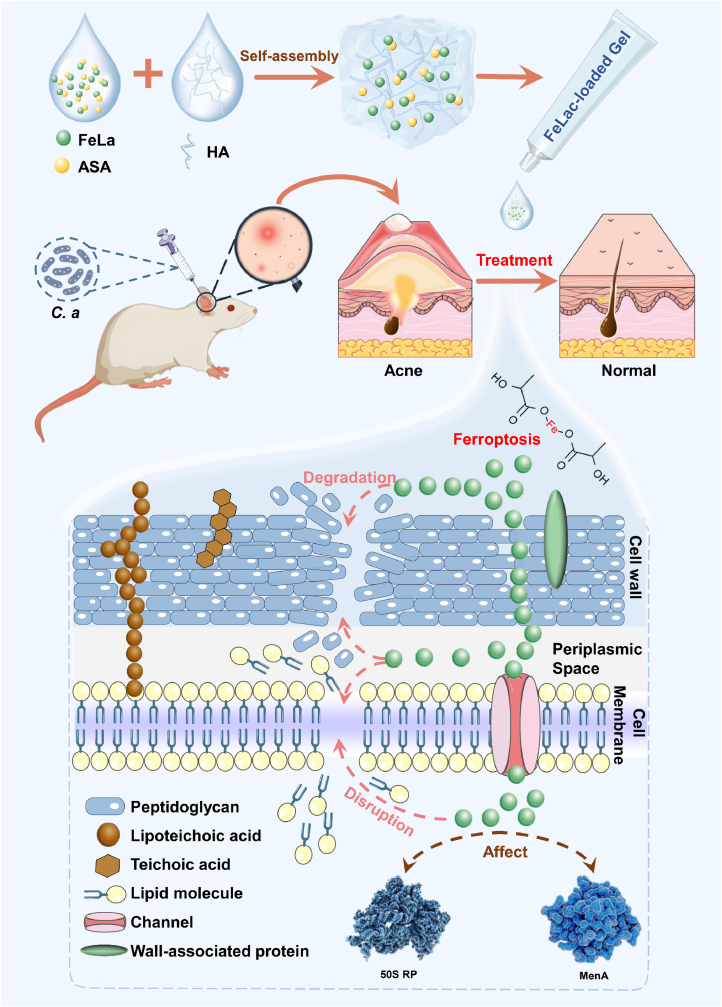
FeLac-loaded hydrogel based on non-canonical ferroptosis for the treatment of acne.

To overcome these challenges, this study investigated the underlying mechanisms of iron-dependent bacterial death under anaerobic conditions and found that direct exposure to ferrous lactate (FeLac) has highly efficient antibacterial effects on *C. acnes*. Based on these findings, this study developed a novel FeLac-loaded hydrogel based on HA to enhance the therapeutic effect while the stability of the system, effectively protect the active ingredients, achieve the characteristics of sustained release of the drug, and robust antibacterial activity. Additionally, FeLac-loaded hydrogel has good biocompatibility and does not cause irritation or sensitization to the skin. In a rat model of *C. acnes*-induced acneiform lesions, topical application of FeLac-loaded hydrogel significantly reduced inflammation and promoted lesion resolution through Fe^2+^ release, achieving therapeutic efficacy comparable to that of common antibiotics (Fig. 1). These findings suggested that FeLac-loaded hydrogel represented a promising and safe alternative to conventional acne therapies and other anaerobic bacteria infections. By providing a non-antibiotic treatment modality (iron-dependent non-canonical ferroptosis), this approach had the potential to mitigate the growing concern of antibiotic resistance, paving the theoretical basis and support for its future clinical application.

## 2 Materials and methods

### 2.1 Strains and chemicals

*Cutibacterium acnes* 6919 used in this study was obtained from the China General Microbiological Culture Collection Center (CGMCC), and cultivated in liquid Gifu Anaerobic Medium (GAM) at 37 °C for 48-72 h. To eliminate the influence of iron ions present in the culture medium, the *C. acnes* cells were rigorously washed prior to the iron compound treatments. Specifically, the cells were harvested by centrifugation, the GAM supernatant was completely discarded, and the bacterial pellets were subsequently resuspended and washed twice in a 0.9% sterile sodium chloride (NaCl) solution. The bacterial suspension was diluted to a concentration of 10^6^ CFU/mL, and all further experiments were performed in a 0.9% NaCl solution. Hyaluronic acid (HA) was purchased from Macklin (Shanghai Macklin Biochemical Technology Co., Ltd., China), with a purity of 97.065%, a glucuronic acid content (on a dry basis) of 46.8%, and a molecular weight of 400–700 kDa. All iron compounds were purchased from Macklin and other chemical agents were analytical grade.

### 2.2 Susceptibility assays to iron compounds

Cells were treated with 200 μM ferrous sulfate (FeSO_4_), ferrous chloride (FeCl_2_), ferrous gluconate (C_12_H_22_O_14_Fe), ferrous fumarate (C_4_H_2_FeO_4_), ferrous lactate (C_6_H_10_FeO_6_), potassium ferrocyanide (K_4_[Fe(CN)_6_]·3H_2_O) and ferric chloride (FeCl_3_), ferric citrate (C_6_H_8_O_7_Fe), iron pyrophosphate (Fe_4_(P_2_O_7_)_3_), iron dextran (FeH_2_O_4_S), potassium ferricyanide (K_3_[Fe(CN)_6_]) at 37 °C for 3 h, respectively. After incubation, cells were collected by centrifugation at 8000×*g* for 5 min and resuspended twice with 0.9% NaCl solution. Susceptibility assays were performed by plating 4 μL drops of serially diluted bacterial suspensions on Gifu Anaerobic Medium Agar (GAMA). Colony-forming units (CFUs) were enumerated after incubation at 37 °C for 48-72 h.

### 2.3 Cell death assay by propidium iodide (PI)

Cells (2 × 10^6^ CFU/mL) in 0.9% NaCl solution were treated with iron compounds at 37 °C for 3 h. After treatment, the bacterial suspension was centrifuged at 8000 rpm for 5 min, and the cell pellets were resuspended in 0.9% NaCl solution. To assess membrane integrity, cells were incubated with 1 μg/mL propidium iodide (PI, ST511, Beyotime Institute of Biotechnology, China) at 4 °C for 30 min in the dark. Stained cells were then analyzed using flow cytometry (Agilent NovoCyte) and fluorescence microscopy (Zeiss Axio Vert A1).

### 2.4 Detection of reactive oxygen species (ROS)

Intracellular ROS production in *C. acnes* following treatment with ferrous compounds was assessed using the fluorescent probe 2′,7′-dichlorodihydrofluorescein diacetate (DCFH-DA) (Sigma-Aldrich, St. Louis, MO, USA). Briefly, *C. acnes* cell suspensions (2 × 10^6^ CFU/mL) were centrifuged at 8000×*g* for 5 mi, washed twice and resuspended in 0.9% NaCl solution to remove residual culture medium. Samples were then incubated with 10 μM DCFH-DA at 37 °C for 30 min in the dark before exposure to iron compounds. ROS generation was subsequently analyzed by using flow cytometry (Agilent NovoCyte) and fluorescence microscopy (Zeiss Axio Vert A1) [24].

### 2.5 Measurement of lipid peroxidation (LPO)

Intracellular LPO levels in *C. acnes* following treatment with FeLac were evaluated using the C11-BODIPY (581/591) probe (D3861, Thermo Fisher Scientific, U.S.), according to Ref. [19]. Briefly, *C. acnes* suspensions (2 × 10^6^ CFU/mL) were exposed to iron compounds for 3 h. After treatment, the bacterial suspension was centrifuged at 8000×*g* for 5 min, and the resulting pellets were washed twice with 0.9% NaCl solution to remove any residual ferrous compounds. The cells were then incubated with 1 μM C11-BODIPY (581/591) at 37 °C for 30 min. Lipid peroxidation levels were subsequently measured by using flow cytometry (Agilent NovoCyte) and fluorescence microscopy (Zeiss Axio Vert A1).

### 2.6 DNA damage assay

Following treatment with ferrous compounds and elution, DNA damage was assessed using a one-step TUNEL apoptosis assay kit (Beyotime Biotechnology, Shanghai, China), according to the manufacturer's instructions. The cells were then analyzed using flow cytometry and fluorescence microscopy.

### 2.7 Detection of intracellular Fe^2+^

Intracellular Fe^2+^ levels in *C. acnes* were assessed using the activatable fluorescent probe FeRhoNox-1 (GC901, Goryo Chemical, Japan). Specifically, the bacteria treated with iron compounds were centrifuged at 8000×*g* for 5 min and washed twice with normal saline. The cells were loaded with FeRhoNox-1 probe (5 μM) and incubated at 37 °C with shaking at 200 rpm for 60 mi. Intracellular Fe^2+^ levels were subsequently analyzed by using both flow cytometry and fluorescence microscopy [25]. Additionally, 2,2′-Dipyripyl probe was utilized as a high affinity Fe^2+^ chelator in this study, samples without the addition of 2,2′-Dipyripyl probe were as the control group.

### 2.8 Scanning electron microscope (SEM) observation

Cell suspensions were collected and treated with iron compounds at 37 °C for 3 h. The samples were then centrifuged at 8000×*g* for 5 mi, washed with 0.9% NaCl solution to remove the iron compounds, and fixed with precooled 2.5% glutaraldehyde (G5882, Sigma-Aldrich, U.S.) at 4 °C for 2 h. After fixation, the cells were washed with 0.9% NaCl and dehydrated with ethanol (ranging from 30% to 100%) for 10 min. The samples were then centrifuged at 8000×*g* for 5 min to remove the ethanol and resuspended twice in isoamyl acetate with a 20-min interval between each resuspension. Finally, all treated samples were coated with gold using a metallizer (Kyoto Shimadzu IC-50) and observed under scanning electron microscopy (SEM, Kyoto Shimadzu SS550) at an accelerating voltage of 15 KV and a magnification of 10000×.

### 2.9 Proteomics analysis

*C. acnes* cells exposed to 0 μM and 200 μM Fe^2+^ for 3 h were sampled, quick-frozen in liquid nitrogen, and then transferred to 5 mL centrifuge tubes. The protein supernatant was collected, and protein concentration was determined using a BCA kit (Beyotime Biotechnology, China) according to the manufacturer's instructions. Trichloroacetic acid was then slowly added to the sample to precipitate the proteins. The precipitated proteins were subjected to trypsin digestion and subsequently analyzed using a mass spectrometer. After data acquisition, the DIA data were processed using the DIA-NN search engine (v.1.8). Tandem mass spectra were searched against the *C. acnes* data concatenated with a reverse decoy database. Further filtering of the search results was performed to obtain high-quality analysis outcomes. Differentially expressed proteins (DEPs) were identified by Fisher's exact test with a p-value <0.05 and a fold change >1.5. DEPs were visualized through a volcano plot and further analyzed using Gene Ontology (GO) annotation, Clusters of Orthologous Groups (COG)/Eukaryotic Orthologous Groups (KOG) annotation, and Kyoto Encyclopedia of Genes and Genomes (KEGG) pathway annotation to determine the functional classification of DEPs in GO terms, COG, or metabolic pathways, as described previously.

### 2.10 Preparation of FeLac-loaded hydrogels

The method proposed by Mo et al. [26] was modified to prepare FeLac-loaded hydrogels using HA as the substrate. In brief, FeLac and L-ascorbic acid (ASA) were dissolved in sterile water under continuous stirring to prepare a FeLac–ASA complex solution with a final concentration of 2 mM. Subsequently, varying amounts of HA powder were gradually added to the solution to achieve final HA concentrations of 1%, 2%, 3%, 4%, and 5% (w/v). The mixtures were stirred thoroughly to ensure complete dispersion and then transferred to a constant-temperature environment at 40 °C. Continuous mixing was maintained using a mechanical overhead stirrer to facilitate gelation. Over time, crosslinking occurred spontaneously, and the solutions transformed into visibly uniform hydrogels, at which point stirring was discontinued.

### 2.11 Characterization of FeLac -loaded hydrogels

#### 2.11.1 Low-field nuclear magnetic resonance

The relaxation time of FeLac -loaded hydrogels was measured using a low-field nuclear magnetic resonance (LF-NMR) analyzer (23.2 MHz, Numag Technology Co., Suzhou, China) [27].

#### 2.11.2 Rheological properties

The rheological behavior of FeLac-loaded hydrogels was analyzed using a rheometer (AR 2000, TA Instruments, United Kingdom) within a frequency range of 1–10 rad/s at 25 °C. A 1 mm gap was maintained between two parallel plates (d = 40 mm). The variations in storage modulus (G′) and loss modulus (G″) as functions of frequency (0.1–10 Hz) were recorded. A strain sweep test was performed at a fixed frequency of 1 Hz within a strain range of 0.1–100% to determine the linear viscoelastic range (LVR), ensuring that all subsequent measurements were conducted within this region. The viscosity change curve was measured at a shear rate of 0.1–10 s^−1^. Additionally, a strain sweep test (0.1–100%) and a stress sweep measurement (1–1000 Pa) were conducted. A creep test was performed within the linear viscoelastic region by applying a constant stress of 10 Pa to the sample for 180 s, followed by stress removal to assess creep recovery over 600 s.

#### 2.11.3 SEM and stability of FeLac-loaded hydrogels

The morphology of hydrogels was visualized via SEM (Kyoto Shimadzu SS550) as described in 2.8. The stability of FeLac-loaded hydrogels was assessed by storing the samples at 4 °C and 25 °C. The morphological changes were recorded through photographic observation.

### 2.12 Ferrous ion release from FeLac-Loaded hydrogel

The release of ferrous ions from FeLac-loaded hydrogel was determined with minor modifications to a previously described method [28]. Briefly, 10 mL of the prepared hydrogel was placed in a dialysis bag (500 Da), which was then submerged in 100 mL of ultrapure water. The system was incubated at 37 °C under agitation at 200 rpm for 12 h. The concentration of ferrous ions in the dialysate was measured every 30 min using the phenanthroline colorimetric method.

### 2.13 Determination of bactericidal properties of FeLac-loaded hydrogel in vitro

The antibacterial activity of FeLac-loaded hydrogel was assessed according to the previously described method with minor modifications [29]. Briefly, 1 mL of the bacterial suspension was mixed with 5 mL of FeLac-loaded hydrogel and incubated at 37 °C for 3 h. The mixture was then spread onto a disposable Petri dish. The bactericidal properties of FeLac-loaded hydrogel were evaluated by calculating the survival rate of *C. acnes* cells following incubation at 37 °C for 72 h.

### 2.14 Skin irritation and sensitization tests sensitization

Within 1 h after depilation of the dorsal skin of rats, the area was examined for signs of skin damage, bleeding, or other abnormalities. If the skin at the depilated site remains intact, disinfect it with 75% alcohol. Subsequently, hydrogel (2 ml) was applied to the site, and immediate photographs were taken for documentation. The rats were then returned to their cages for unrestricted movement, and take photographs at 0, 4, 24, 48, 72 and 96 h for documentation. Simultaneously, signs of erythema, edema, or other adverse reactions at the application site were recorded, along with monitoring for systemic allergic reactions, such as asthma or ataxia.

### 2.15 Subcutaneous biocompatibility studies of FeLac-loaded hydrogel

The biodegradation and biocompatibility of FeLac-loaded hydrogel were evaluated by subcutaneously implanting sterilized hydrogels in rats [30]. Briefly, 2 mL of hydrogel was injected subcutaneously into rats. Subsequently, photographs were taken of the injected tissue at 0.5 h and 24 h post-injection and histological examination using hematoxylin and eosin (HE) staining was conducted to assess the inflammatory response in the skin tissue.

### 2.16 In vitro cytotoxicity

Cytocompatibility of the hydrogels was evaluated via both direct contact and extract-based assays, following a modified protocol adapted from Wang et al. [31]. To prepare the extraction medium, 1 mL of the sterilized hydrogel was immersed in 10 mL of sterile physiological saline and incubated at 37 °C for 24 h. Human skin fibroblasts (HSFs) in the exponential growth phase were harvested by trypsinization and resuspended in complete culture medium at a density of 2.5 × 10^3^ cells/mL. The cell suspension was seeded into 24-well plates and incubated at 37 °C in a humidified atmosphere containing 5% CO_2_ for 24 h to facilitate optimal cellular adhesion. After adhesion, the culture medium was removed and replaced with fresh medium supplemented with 50 μL of either intact sterilized hydrogels (direct contact group) or the corresponding extraction medium (extract group). Cells cultured in standard complete medium served as the control group. After continuous co-incubation for 24, 72, and 120 h, cell viability was quantitatively assessed utilizing a Cell Counting Kit-8 (CCK-8) assay according to the manufacturer's protocol. All experiments were performed in triplicate to ensure reproducibility.

### 2.17 In vitro skin penetration

The in vitro skin permeation study was performed using vertical Franz diffusion cells following a slightly modified protocol based on that reported by Šmejkalová et al. [32]. Excised abdominal skin from 1.5-month-old male Bama miniature pigs (Beijing Fuhao Experimental Animal Breeding Center, Beijing, China; Animal Quality Certificate No. 110344251100181884) was used as the permeation barrier. The skin samples were trimmed to a uniform thickness of 0.75 mm and pre-equilibrated in physiological saline at 2–8 °C prior to experimentation. To mimic a compromised skin barrier, superficial cross-shaped incisions (∼5 mm in length) were carefully introduced using a sterile scalpel, ensuring disruption of the stratum corneum without affecting the underlying dermis. The prepared skin was then mounted between the donor and receptor compartments of the Franz diffusion cells, with the stratum corneum facing the donor chamber. Subsequently, 0.5 mL of the hydrogel formulation was applied to the donor chamber and diluted with physiological saline to a final volume of 2 mL. The receptor chamber was filled with physiological saline and maintained at 32 ± 1 °C under continuous magnetic stirring at 350 rpm to simulate physiological skin conditions. At predetermined time intervals (every 1 h), samples were withdrawn from the receptor compartment, and the concentration of ferrous ions was quantified using the phenanthroline colorimetric method.

### 2.18 Establishment and treatment of a rat model of *C. acnes*-induced acneiform lesions

Animal experiments adhered to ARRIVE guidelines and were approved by the Laboratory Animal Welfare and Ethical Review Committee of Henan Institute of Science and Technology (Protocol #LLSC2023038). The rat model of *C. acnes*-induced acne-like lesions was established as described previously, with minor modifications [[33], [34], [35]]. Wistar male rats used in the study were acclimatized for one week prior to the experiment (12 h light/12 h dark cycle with ad libitum access to food and water). Subsequently, the rats were weighed and numbered, and acne-like symptoms in a rat model of *C. acnes*-induced ear. A 0.5 mL solution of 100% oleic acid (Shanghai Macklin Biochemical Technology Co., Ltd., China) was applied using a glass rod to a 2 cm × 2 cm area at the opening of the right ear canal. The next day, 50 μL of *C. acnes* bacterial suspension was injected subcutaneously into the right ear pinna for seven consecutive days. After completing the model induction, two rats were randomly selected for macroscopic morphological assessment, including skin color, swelling severity, ear thickness, sebaceous gland diameter, and lesion characteristics (comedones, papules, and pustules) to confirm successful model establishment. The successfully modeled rats were then randomly assigned into six groups (n ≥ 6 per group): NaCl treatment group with daily application of 0.9% NaCl to the ears. HA treatment group with daily application of HA hydrogel to the ears. HA-ASA treatment group with daily application of HA-ASA hydrogel to the ears. HA-Fe treatment group with daily application of FeLac-loaded hydrogel to the ears. Clindamycin treatment group with daily application of Clindamycin Phosphate Gel (Yunnan Phyto Pharmaceutical Co., Ltd, China) to the ears. Adapalene treatment group with daily application of Adapalene Gel (Laboratoires Galderma, France) to the ears. Blank treatment group with rats receiving parallel administration of 0.9% NaCl. The application dosage for all treatments was standardized at 0.02 mL/cm^2^ per day.

On days 12, rats from each group were euthanized under anesthesia to collect ear tissue and blood samples. Levels of TNF-α, IL-6, and IL-1β were quantified using ELISA kits following the manufacturer's instructions. Additionally, ear tissue samples were subjected to hematoxylin and eosin (HE) staining for histological evaluation.

### 2.19 Statistical analysis

All experiments were performed in triplicate. Statistical analysis was performed using OriginPro 2024 (OriginLab Corporation., Northampton, USA) and Statistical Package for Social Science (SPSS 27, SPSS Inc., Chicago, IL, USA). One-way analysis of variance (ANOVA) was used to compare differences between the treatments. Difference was considered statistically significant at *P* < 0.05.

## 3 Results

### 3.1 The bactericidal effects of iron compounds on *C. acnes*

**Fig. 2:**
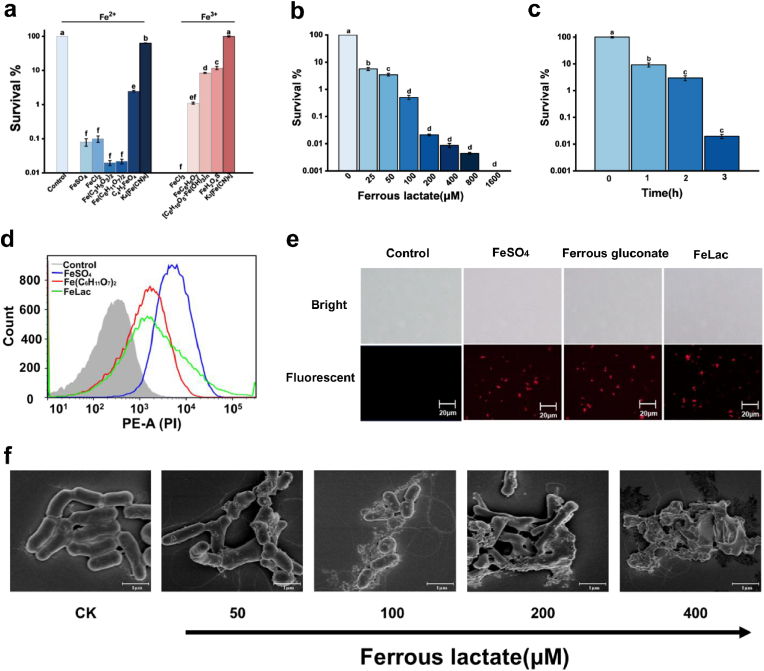
Bactericidal effects of iron compounds on *C. acnes*. (a) Survival of *C. acnes* cells after exposure to eleven iron compounds at 200 μM for 3 h. (b) Dose-dependent effects of FeLac on *C. acnes* for 3 h. (c) Time-dependent effects of FeLac on *C. acnes* at 200 μM. (d) Flow cytometry analysis of propidium iodide (PI)-stained *C. acnes* cells after exposure to 200 μM FeSO_4_, FeLac, and ferrous gluconate for 3 h. (e) Fluorescence microscopic images of PI-stained *C. acnes* cells after exposure to 200 μM FeSO_4_, FeLac, and ferrous gluconate for 3 h. (f) SEM images of *C. acnes* cells exposed to 50, 100, 200, 400 μM FeLac.

Bactericidal assays demonstrated that nine 200 μM iron compounds demonstrated significant bactericidal effects, with FeLac and FeCl_3_ achieving a kill rate exceeding 99.98% (Fig. 2a). Given the known safety concerns associated with FeCl_3_, including potential chloride homeostasis disruption and corrosiveness, which may cause irritation to the skin and respiratory tract [25,36,37], FeLac was selected for further investigations. FeLac exhibited a dose- and time-dependent bactericidal effect on *C. acnes* (Fig. 2b–c), the optimal bactericidal condition was determined as treatment with 200 μM FeLac for at least 3 h, which resulted in a kill rate exceeding 99.9%. Flow cytometry and fluorescence microscopy analysis revealed a marked increase in red fluorescence intensity following FeLac treatment, confirming cell membrane disruption, ruling out a viable but non-culturable state (Fig. 2d–e). SEM analysis further confirmed membrane budding, rupture, and leakage of intracellular contents, ultimately resulting in cell lysis with increasing FeLac concentrations (Fig. 2f).

### 3.2 FeLac induced intracellular iron overload in *C. acnes*

**Fig. 3:**
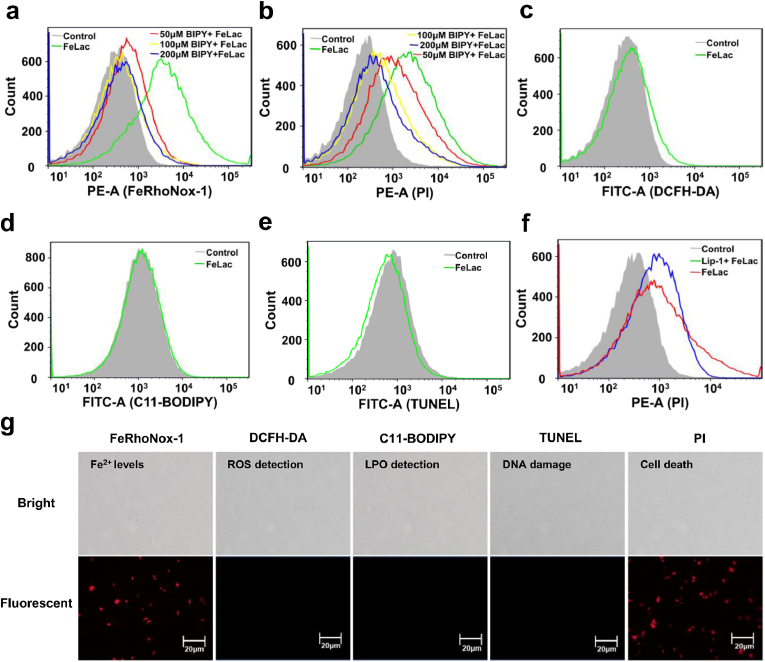
FeLac induced non-canonical ferroptosis in *C. acnes*. Flow cytometry analysis of intracellular Fe^2+^ levels (FeRhoNox-1) (a), cell death (PI) (b), ROS production (DCFH-DA) (c), lipid peroxidation production (C11-BODIPY) (d), DNA damage (TUNEL) (e), and the effect of the ferroptosis inhibitor Lip-1 on cell death (PI) (f) in *C. acnes* cells exposed to 200 μM FeLac. (g) Fluorescence microscopy observation of Fe^2+^ levels, ROS production, lipid peroxidation production, DNA damage, and the effect of the ferroptosis inhibitor Lip-1 on cell death.

Flow cytometry and fluorescence microscopy analyses demonstrated a significant increase in intracellular Fe^2+^ levels with FeLac exposure, and the exogenous addition of the Fe^2+^ chelator 2,2′-bipyridine effectively reduced intracellular Fe^2+^ accumulation, mitigated FeLac-induced cell death, indicating that FeLac-induced intracellular iron overload as a key bactericidal mechanism (Fig. 3a–b).

### 3.3 FeLac triggered a non-canonical ferroptosis in *C. acnes*

To investigate whether FeLac induced ferroptosis in *C. acnes*, intracellular ROS, LPO, and DNA damage were assessed. Notably, there was no significant effect on the level of intracellular ROS, LPO and DNA damage after treated with FeLac (Fig. 3c–g). Furthermore, Liproxstatin-1 (Lip-1), a potent ferroptosis inhibitor, could suppress lipid peroxidation and the Fenton reaction [38]. As shown in Fig. 2f, Lip-1 failed to prevent FeLac-induced cell death and showed no mitigation effect.

### 3.4 Proteomics analysis of FeLac-Induced ferroptosis in *C. acnes*

**Fig. 4:**
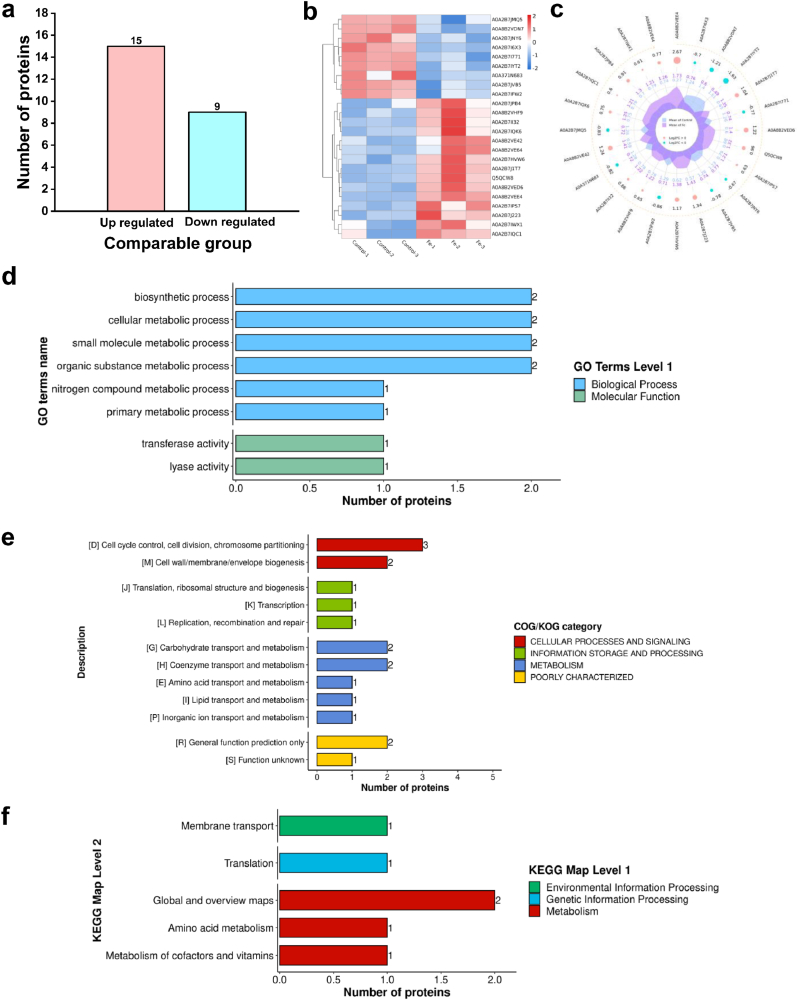
Proteomic analysis of *C. acnes* cells under FeLac stress. (a) Differentially expressed genes (DEGs) in *C. acnes* cells after Fe^2+^ exposure. (b-c) Volcano and radar plots of DEGs. (d-f) Gene ontology (GO) annotation, Clusters of Orthologous Groups (COG)/Eukaryotic Orthologous Groups (KOG) classification, and Kyoto Encyclopedia of Genes and Genomes (KEGG) pathway analysis of DEGs.

To further elucidate the lethal effects of FeLac on *C. acnes*, a comparative proteomic analysis was performed between FeLac-exposed cells (200 μM) and control cells incubated without FeLac (Fig. 4) (Tables S1–4; see Supporting Information). Proteomics identified 1683 proteins, with 24 DEPs, including 15 upregulated (>1.5-fold) and 9 downregulated (<1.5-fold) proteins.

Specifically, the analysis showed minimal changes in proteins associated with fatty acid metabolism, ROS response, DNA damage, and iron-sulfur cluster synthesis. However, significant alterations were observed in specific targets: the downregulation of 50S ribosomal proteins (A0A2B7I771) and imidazole glycerol phosphate dehydratase (IGPD) (A0A2B7JVB5), alongside the upregulation of RNA polymerase (A0A2B7J223) and 1,4-dihydroxy-2-naphthoate octaprenyltransferase (MenA) (A0A2B7IPS7). FeLac exposure also resulted in a significant upregulation of multiple proteins involved in cell wall degradation (A0A2B7IWX1, A0A8B2VHF9, A0A2B7I6X3, A0A2B7IQC1, A0A2B7J1T7, A0A8B2VE42, A0A8B2VED6, A0A8B2VEE4). Furthermore, functional annotation revealed that FeLac exposure affected metabolic pathways, including biosynthesis, nitrogen metabolism, and cofactor/vitamin metabolism—most of which have been implicated in the regulation of cell death [39,40].

### 3.5 Preparation and characterization of FeLac-loaded hydrogels

**Fig. 5:**
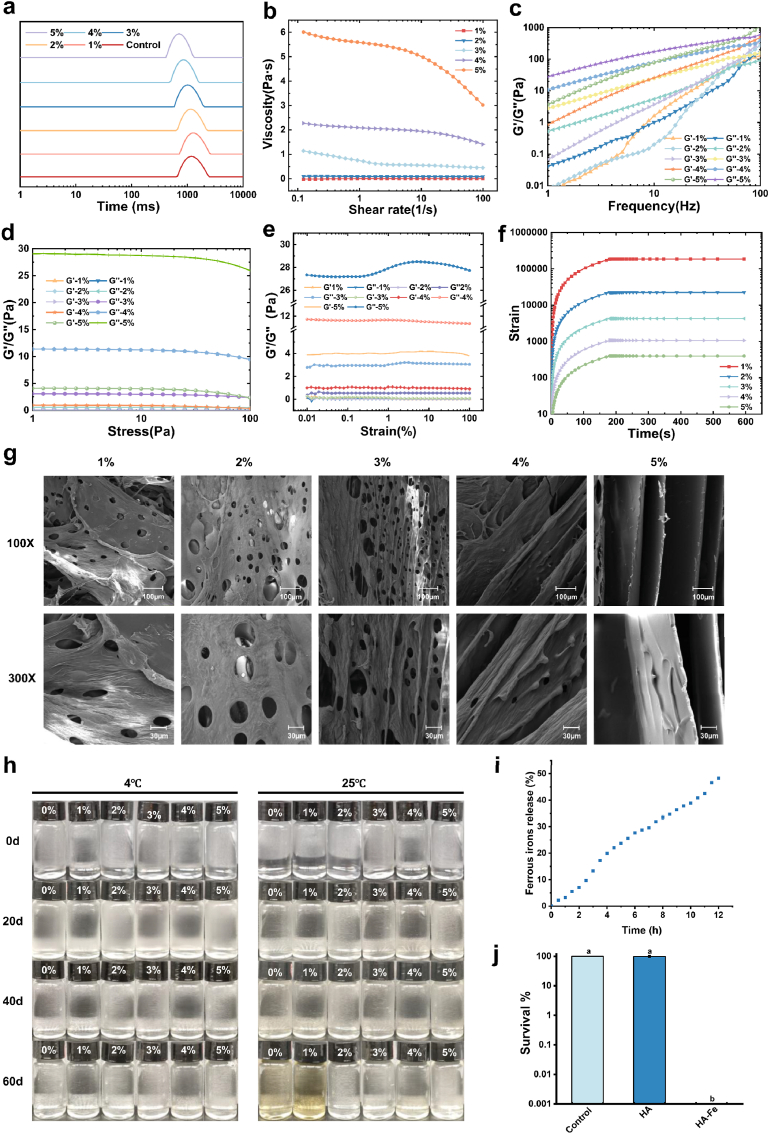
Preparation and characterization of FeLac-loaded hydrogels. (a) Low-field nuclear magnetic resonance (LF-NMR) analysis of hydrogels with different concentrations of hyaluronic acid (HA: 1%, 2%, 3%, 4%, and 5%). Viscosity curves (b), Frequency sweep curves (c), Stress curves (d), Strain curves (e), Creep curves (f) of hydrogels with different concentrations of hyaluronic acid (HA: 1%, 2%, 3%, 4%, and 5%). (g) SEM images of hydrogels with different concentrations of hyaluronic acid (HA: 1%, 2%, 3%, 4%, and 5%). (h) Storage stability of hydrogels with different concentrations of hyaluronic acid (HA: 1%, 2%, 3%, 4%, and 5%) under room temperature (25 °C) and refrigerated conditions (4 °C). (i) Fe^2+^ release profile of 3% HA hydrogel. (j) In vitro antibacterial activity of FeLac-loaded hydrogel (HA: 3%).

To determine the optimal ratio of HA within the hydrogel system, characterization of FeLac-loaded hydrogels at varying concentrations of HA was conducted. LF-NMR analysis showed that increasing HA concentration reduced T2 relaxation times, indicating enhanced hydration and improved network stability (Fig. 5a). Rheological analysis revealed the viscosity of the hydrogel decreased with increasing shear rate, confirming their shear-thinning behavior and pseudoplastic fluid characteristics (Fig. 5b). As shown in Fig. 4c, at low frequencies, the hydrogel showed viscous behavior (G'' > G′), transitioning to more elastic properties at higher frequencies. The G″/G′ ratio reflects the viscoelastic balance of the hydrogel, where a lower value indicates a more dominant elastic (solid-like) behavior and a more stable crosslinked network structure [41]. Notably, this ratio progressively decreased with increasing HA content, indicating a shift toward more elastic-dominated behavior and improved network integrity (Fig. 5d and e). Moreover, low-concentration hydrogels had poor mechanical stability, while higher concentrations showed better stability and persistence (Fig. 5f). As shown in Fig. 5g, FeLac-loaded hydrogel is composed of interconnected sheet-like structures. The 3% HA group exhibited a uniform porous microstructure and well-formed polymer network, features conducive to efficient hydration and drug-loading capacity. As the Fe^2+^ content in the system decreases, larger and smoother bundles are formed, indicating that the interwoven network is crosslinked by Fe^2+^. Stability testing indicated that all hydrogels maintained excellent physical integrity when stored at 4 °C. However, formulations with lower HA concentrations exhibited Fe^2+^ oxidation and visible discoloration when stored at 25 °C (Fig. 5h). Based on these findings, 3% HA was selected for the preparation of FeLac-loaded hydrogels.

Iron ion release assays showed that a sustained and time-dependent release of Fe^2+^ from the hydrogel, with approximately 48.3% of the total Fe^2+^ content released within 12 h (Fig. 5i). As shown in Fig. 5j, the HA hydrogel alone did not exhibit significant bactericidal activity. In contrast, FeLac-loaded hydrogel effectively killed pathogenic *C. acnes*.

### 3.6 Biocompatibility and transdermal permeation of FeLac-loaded hydrogel

**Fig. 6:**
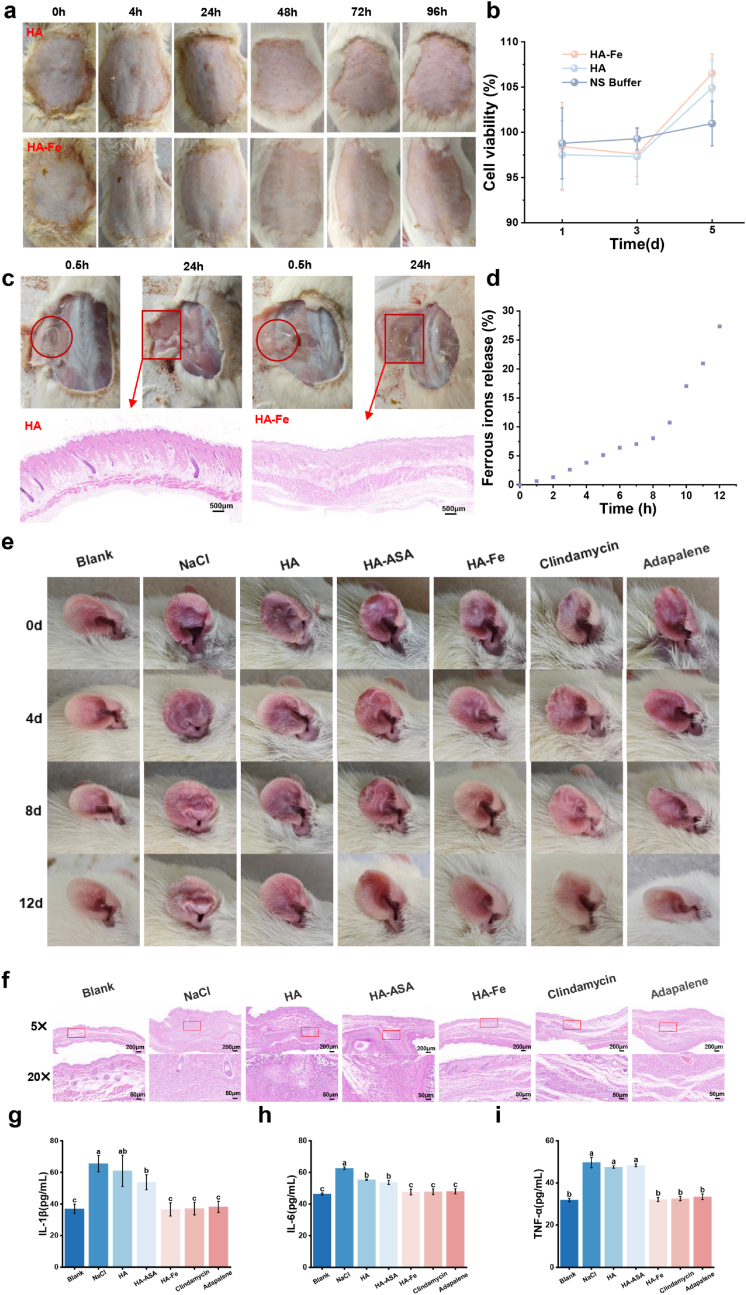
Biocompatibility, skin penetration evaluation and therapeutic properties of FeLac-loaded hydrogel. (a) Skin irritation and sensitization potential of FeLac-loaded hydrogel (HA: 3%). (b) Cell viability of human skin cells after treatment with different concentrations of FeLac-loaded hydrogel. (c)Biocompatibility of FeLac-loaded hydrogel in subcutaneous implantation. (d)skin penetration of FeLac-loaded hydroge evaluated using Franz diffusion cell system. (e) Therapeutic efficacy of FeLac-loaded hydrogel on acne in rat ear. (f) Histology observation of HE staining of ear tissues in rat after treatments. Changes in inflammatory cytokines IL-1β (g), IL-6 (h), and TNF-α (i) in treated rats.

Skin irritation/sensitization tests demonstrated that topical application of the FeLac-loaded hydrogel did not induce noticeable erythema, edema, or other adverse skin reactions throughout the observation period, nor did it trigger any systemic allergic responses (Fig. 6a). Subcutaneous implantation further revealed that the hydrogel gradually diffused and was absorbed by the surrounding tissue without triggering severe inflammatory responses within 24 h post-injection (Fig. 6c). Histological evaluation via H&E staining confirmed that skin appendages remained intact, collagen fiber bundles appeared well-organized, and tissue disruption was minimal. Furthermore, the in vitro cytocompatibility of the hydrogel was evaluated using human skin fibroblasts (HSFs) (Fig. 6b). The results indicated that after 3 days of incubation in the hydrogel extraction medium, there was no significant difference in cell viability compared to the control group, with >98% of the HSFs remaining viable. Notably, by day 5, the FeLac-loaded hydrogel was found to promote cell proliferation, with cell viability exceeding 100%. Collectively, these findings from skin irritation/sensitization tests, in vivo tissue compatibility, and in vitro cytocompatibility assays robustly demonstrate the excellent overall biocompatibility of the formulation. Additionally, ex vivo transdermal permeation studies were conducted to evaluate its delivery performance. The results showed that approximately 28.5% of Fe^2+^ permeated through the skin within 12 h(Fig. 6d). Although this permeation rate was lower than that observed in the in vitro release study, it still indicates effective transdermal delivery capability. This difference is likely attributable to fundamental disparities in transport mechanisms, as intact skin presents a highly organized lipid barrier and active metabolic processes that restrict molecular diffusion [42]. The FeLac-loaded hydrogel exhibited high biocompatibility and demonstrated effective skin penetration, supporting its suitability for topical application.

### 3.7 Therapeutic effect of FeLac-loaded hydrogel

The therapeutic efficacy of FeLac-loaded hydrogel against *C. acnes* infection was evaluated using a rat model of *C. acnes*-induced acne-like lesions. As shown in Fig. 6e, on the onset of therapy (Day 0), the ears of infected rats displayed noticeable acne symptoms, including pronounced swelling, rough epidermis, dark red discoloration, and visible pustules and papules. Following eight days of treatment with FeLac-loaded hydrogel, the acne symptoms significantly improved, with a marked reduction in redness and inflammation. By Day 12, the lesions had almost completely resolved, with the treated ears closely resembling those of the blank control group, indicating excellent therapeutic efficacy. Clindamycin and adapalene, used as positive controls, also demonstrated therapeutic effects comparable to those of FeLac-loaded hydrogel.

Histological analysis further supported these findings (Fig. 6f). HE staining revealed that intact auricular tissue in the blank group, and no significant abnormalities. In contrast, NaCl-treated rats exhibited severe auricular tissue destruction, thickened stratum corneum, hyperkeratosis, and pronounced inflammation. Notably, FeLac-loaded hydrogel, clindamycin, and adapalene treatments nearly restored normal skin architecture.

Given that acne is an immune-driven condition critically mediated by macrophages and neutrophils, evaluating systemic inflammation through blood cytokine levels is essential [43]. As shown in Fig. 6g–i, after 14 days of treatment with sodium chloride, HA hydrogel, and HA-ASA hydrogel, blood samples from treated rats still exhibited elevated levels of the pro-inflammatory cytokines IL-1β, IL-6, and TNF-α. In contrast, treatment with FeLac-loaded hydrogel, clindamycin, or adapalene significantly reduced systemic pro-inflammatory cytokine levels. Notably, these quantitative reductions are in strong agreement with the observed morphological improvements, including decreased swelling and erythema, as well as the restoration of normal skin architecture in H&E staining (Fig. 6e and f). In summary, these findings demonstrate that the FeLac-loaded hydrogel demonstrated significant therapeutic efficacy in treating *C. acnes*-induced acneiform lesions, effectively reducing both local and systemic inflammation. Importantly, the integration of qualitative morphological observations with quantitative immunological data enables a more comprehensive and reliable assessment of treatment efficacy.

## 4 Discussion

The development of acne vulgaris is primarily attributed to abnormal follicular keratinization and excessive sebum production, which lead to the overgrowth of *C. acnes* (a lipophilic anaerobic bacterium) and the loss of microbial balance among its subtypes [44]. Currently, the most common approach for acne treatment involves the topical or systemic administration of single or combination antibiotics (benzoyl peroxide, clindamycin, erythromycin, dapsone, and minocycline) to reduce the concentration of *C. acnes* colonization [45]. However, classic antibiotic therapy has become less effective because the ability of *C. acnes* to evolve natural AMR and form biofilms that tolerate antibiotics [7,46]. Therefore, research into alternative antimicrobial agents for acne treatment is increasingly important. To the best of our knowledge, this study is the first to utilize FeLac-loaded hydrogel as an alternative to conventional antibiotics for the treatment of anaerobic bacteria infection and acne vulgaris.

Kohanski et al. [47] proposed that, despite targeting distinct upstream pathways, various antibiotics ultimately converge on shared downstream bacterial death mechanisms. These included the degradation of iron-sulfur cluster proteins and the expression of iron-related genes, resulting in elevated intracellular free iron levels. Free iron acts as a key reactant in lethal damage, producing highly destructive ROS through the Fenton reaction to kill bacteria. Theoretically, directly targeting the downstream pathways of the bactericidal mechanism (free iron) can bypass mutations in upstream targets, thereby achieving effective bactericidal activity and mitigating the emergence of antibiotic resistance. Our previous research demonstrated that exposure to iron compounds under non-culture conditions effectively induced cell death in *Staphylococcus aureus*, *Escherichia coli*, *Pseudomonas aeruginosa* and *Candida albicans* [19,[24], [25], [26]]. This effect was attributed to iron-stimulated ROS generation, which specifically triggered the occurrence ferroptosis, and also exhibited significant bactericidal activity against multidrug-resistant bacteria. However, Kohanski's hypothesis has been questioned, as antibiotics could retain their bactericidal activity under hypoxic conditions, despite the inability of cells to form ROS [48,49]. Given that anaerobic bacteria possess unique oxidative stress systems and do not accumulate ROS, it remains unclear whether iron compounds can still effectively induce cell death in these bacteria [50]. Interestingly, we found that exposure to FeLac efficiently killed *C. acnes* through ROS-independent pathways (Fig. 2, Fig. 3). Unlike ferroptosis in aerobic organisms and mammalian cells, FeLac-induced cell death in *C. acnes* proceeded without characteristic hallmarks of oxidative stress (ROS, LPO, or DNA damage), revealing an alternative death mechanism in anaerobic bacteria [51]. This implies that mechanisms by which Fe^2+^ induces microbial ferroptosis are not limited to the well-known oxidative stress pathways, leading to lipid peroxidation, but also involve other pathways independent of ROS.

The proteomics analysis revealed that the response to FeLac differs fundamentally from typical ferroptosis, as evidenced by the lack of significant changes in fatty acid metabolism, ROS response, DNA damage, and iron-sulfur cluster synthesis [19,[24], [25], [26]]. Instead, the observed downregulation of 50S ribosomal proteins points to a disruption in bacterial translation, a known vulnerability targeted by multiple antibiotics [52]. The concurrent upregulation of RNA polymerase may reflect a compensatory mechanism aimed at maintaining essential gene transcription despite this ribosomal disruption [53]. Furthermore, the upregulation of MenA, a key enzyme in bacterial vitamin K2 biosynthesis, suggests an impact on electron transfer within the anaerobic respiratory chain. Notably, the proteomic findings corroborate our SEM observations of compromised bacterial cell morphology. The observed downregulation of IGPD—an enzyme catalyzing a key step in histidine biosynthesis implicated in biofilm formation and bacterial virulence [54,55]—alongside the significant upregulation of cell wall degradation proteins, aligns with the physical cell wall disruption and biofilm degradation of *C. acnes* following Fe^2+^ treatment. Disruption of cell wall integrity can lead to increased susceptibility to external stressors, including antimicrobial agents and host immune defenses [56], which may potentially reduce the occurrence of AMR [46,57]. Collectively, these findings indicate that Fe^2+^ exposure did not directly produce ROS or induce changes in related pathways under anaerobic conditions. Rather, lethality most likely resulted from translational inhibition, impaired protein synthesis, biofilm degradation, and cell wall disruption. Additionally, future studies will focus on developing alternative evaluation strategies to investigate antimicrobial resistance under nutrient-limited or host-mimicking microenvironments. Ultimately, such efforts may open new avenues for the design and development of novel antibacterial agents and bioactive compounds capable of overcoming antimicrobial resistance (AMR).

Furthermore, *C. acnes* (particularly phylotype IA1) was found to stimulate inflammation through both innate (Toll-like receptors, TLRs) and adaptive immunity (IL-17A and IFN-γ secreted by CD4^+^ T cells). It promotes the production of metalloproteinases that degrade the extracellular matrix (ECM), thereby leading to scar and dyspigmentation formation [58]. To counteract these effects, we developed a FeLac-loaded hydrogel utilizing hyaluronic acid (HA) as a biocompatible carrier and ASA as an antioxidant stabilizer. As a fundamental ECM component, HA is crucial for tissue hydration, structural stability, and cellular signaling [59]. Crucially, its biological activity is highly molecular-weight (MW) dependent: low-MW HA (<200 kDa) acts as a pro-inflammatory damage-associated molecular pattern (DAMP), whereas medium-to high-MW HA exerts anti-inflammatory and immunosuppressive effects [60,61]. Additionally, studies indicate that while HA with a molecular weight of 400–2500 kDa forms a highly entangled network, its structural stability inversely correlates with increasing molecular weight [62]. In this study, HA with a molecular weight of 400–700 kDa was employed to avoid the pro-inflammatory effects associated with low-MW fragments while providing favorable rheological properties for hydrogel formation, maintaining tissue hydration and structural stability, and thereby making it particularly suitable for topical acne treatment [63,64]. The stability of the hydrogel is further fortified by its unique crosslinking mechanisms. Previous studies indicate that combining cationic compounds with HA enhances overall system stability [65]. In the presence of metal ions, as the carboxyl groups of HA(glucuronic acid) increase, the hydrogel not only forms some intermolecular hydrogen bond networks, but also some weak hydrogen bonds of the HA hydrogel can be further uniformly crosslinked through another part of metal-carboxylate interactions, enhancing stability and protects the metal ions in the system from oxidation(Fig. 5) [66]. LF-NMR and rheological analysis revealed shear-thinning behavior and self-healing properties, which facilitated better diffusion and drug release, thereby providing effective coverage and protection within tissues [67,68]. Such results were consistent with those of the SEM. In vitro release experiments indicated that the hydrogel prolonged the residence time of Fe^2+^ in the hydrogel network, while slowing down the degradation of the hydrogel, resulting in sustained Fe^2+^ release [69]. According to the “500 Da rule”, compounds with a molecular weight below 500 Da are permissible for skin absorption, suggesting the potential of FeLac-loaded hydrogel for effective transdermal absorption based on time-controlled release, which enhances bioactivity [70]. Notably, the bare HA hydrogel did not exhibit inherent bactericidal activity, a limitation potentially linked to its molecular weight and the local production of hyaluronidase by *C. acnes* [71,72]. In stark contrast, the incorporation of FeLac transforms the hydrogel into a highly effective antimicrobial platform, driving bacterial clearance through the induction of iron-dependent, non-canonical ferroptosis. Biocompatibility, transdermal permeation and therapeutic efficacy experiments confirmed that the composite hydrogel not only exhibited excellent biocompatibility but also demonstrated promising therapeutic potential in treating *C. acnes* infection and resolution of acne lesions(Fig. 6). This effect is likely mediated by the sustained release and effective skin permeation of Fe^2+^, achieving antibacterial efficacy comparable to that of conventional antibiotics. To fully realize the translational potential of FeLac-based topical therapies, future studies must investigate whether this formulation directly modulates sebaceous gland activity and lipid secretion. Beyond its antimicrobial action, the Fe^2+^ confers pleiotropic biological benefits. Accumulating evidence suggests that Fe^2+^ can inhibit Ca^2+^ influx by modulating the transient receptor potential vanilloid 1 (TRPV1) channel, thereby reducing nociceptive signaling and attenuating inflammation associated with TRPV1 activation [73]. Furthermore, Fe^2+^ plays a critical role in activating prolyl-4-hydroxylase (P4H), a key enzyme required for collagen biosynthesis, and thus contributes to tissue regeneration and the maintenance of connective tissue integrity [74,75]. These mechanistic insights are consistent with our experimental observations, in which the hydrogel significantly promoted fibroblast proliferation (Fig. 6b). Our additional results demonstrate that FeLac exhibits potent antibacterial activity against *Staphylococcus epidermidis*(RP62A), an opportunistic skin commensal implicated in acne-associated inflammation(Appendix S1; see Supporting Information) [76]. This broad antimicrobial profile is especially advantageous for preventing secondary infections and managing the complex microbial environment of compromised skin barriers, underscoring its potential as a targeted, non-antibiotic approach for controlling infection and inflammation in acne-prone skin.

Collectively, these findings indicate that the FeLac-loaded hydrogel can effectively mitigate excessive inflammation in early-stage acne lesions while simultaneously promoting tissue repair. This dual functionality may help preserve extracellular matrix homeostasis and reduce the risk of dyspigmentation and scar formation. Therefore, the FeLac-loaded hydrogel represents a promising non-antibiotic therapeutic strategy for infection control, further highlighting its substantial translational potential for dermatological, cosmetic, and broader biomedical applications.

## 5 Conclusion

FeLac-loaded hydrogel offers a novel and effective strategy for treating *C. acnes* and other anaerobic bacterial infections, addressing key limitations of traditional antibiotic therapies. Unlike conventional antibiotics, FeLac-loaded hydrogel not only effectively targets *C. acnes* but also promotes skin repair, prevents scar formation, and inhibits pigmentation by modulating the functions of various skin cells. These results support the potential of FeLac-loaded hydrogel as a non-antibiotic therapeutic platform for acne vulgaris and related anaerobic infections. Mechanistically, this study also revealed that Fe^2+^-induced ferroptosis operates through a ROS-independent mechanism under anaerobic conditions, expanding the understanding of ferroptosis beyond classical oxidative paradigms. Our findings provide insights into the role of the ferroptosis field in anaerobic bacteria and also offer new supplements into Kohanski's theory. Future studies should focus on elucidating the detailed molecular mechanisms underlying these effects, investigating the influence of FeLac-loaded hydrogels on sebum secretion, and validating these findings using advanced three-dimensional human skin equivalents. Such investigations will further support the translational and clinical potential of this system in dermatology.

## Funding sources

This work was financially supported by the 10.13039/501100001809National Natural Science Foundation of China (Grant No. 32272278 and Grant No. 32302028), Key R&D Projects of Shaanxi Province (No. 2024NC-YBXM-176), and Innovation Capability Support Program of Shaanxi Province (Program No. 2023-CX-TD-61).

## CRediT authorship contribution statement

**Rui Wang:** Conceptualization, Investigation, Writing – original draft. **Haizhen Mo:** Funding acquisition, Methodology, Writing – review & editing. **Liangbin Hu:** Conceptualization, Funding acquisition, Writing – review & editing. **Jintae Lee:** Investigation, Writing – review & editing. **Lishan Yao:** Funding acquisition, Writing – review & editing. **Shurui Peng:** Methodology, Writing – review & editing. **Min Zhang:** Data curation, Methodology. **Wei Zhou:** Resources, Writing – review & editing. **Hongbo Li:** Resources, Writing – review & editing. **Jiayi Zhang:** Data curation, Writing – review & editing.

## Declaration of competing interest

The authors declare that they have no known competing financial interests or personal relationships that could have appeared to influence the work reported in this paper.

## Data Availability

Data will be made available on request.
