# Supplementary material for: Ferrous lactate-loaded hydrogels induce iron-dependent non-canonical ferroptosis in Cutibacterium acnes: a novel therapeutic strategy for acne vulgaris
Source: Mater Today Bio. 2026 Apr 29;38:103174. doi: 10.1016/j.mtbio.2026.103174 (PMC13148001; doi:10.1016/j.mtbio.2026.103174)
Supplement: Multimedia component 1 [file mmc1.docx]

**Appendix S1 Antibacterial activity of ferrous lactate against *Staphylococcus epidermidis***

**1.Materials and Methods**

**1.1 Strains and chemicals**

*Staphylococcus epidermidis* 35984 (RP62A) used in this study was obtained from the American Type Culture Collection (ATCC). Bacterial cells were cultured in liquid tryptic soy broth (TSB) at 37℃ with shaking at 200 rpm until reaching the mid-logarithmic growth phase. To eliminate the influence of iron ions present in the culture medium, the *S. epidermidis* cells were rigorously washed prior to the iron compound treatments. Specifically, the cells were harvested by centrifugation, the TSB supernatant was completely discarded, and the bacterial pellets were subsequently resuspended and washed twice in a 0.9% sterile sodium chloride (NaCl) solution. The bacterial suspension was diluted to a concentration of 106 CFU/mL, and all further experiments were performed in a 0.9% NaCl solution*.*

**1.2 Ferrous lactate treatment and antibacterial assay**

For susceptibility profiling, cells were treated with different concentrations of ferrous lactate (Shanghai Macklin Biochemical Technology Co., Ltd.,) at 37℃ for 3 h, respectively. After incubation, cells were collected by centrifugation at 8000 × g for 5 min and resuspended twice with 0.9% NaCl solution. Susceptibility assays were performed by plating 4 µL drops of serially diluted bacterial suspensions on tryptic soy agar (TSA). Colony-forming units (CFUs) were enumerated after incubation at 37℃ for 12-24h. All experiments were performed in triplicate to ensure reproducibility.

**2 Results**

**2.1 Antibacterial activity of FeLac against *Staphylococcus epidermidis***

The bactericidal efficacy of FeLac was further evaluated against *Staphylococcus epidermidis* (RP62A), a prominent opportunistic pathogen found in the cutaneous microbiome. After a 3-hour exposure to 200 μM FeLac, near-complete eradication of the bacterial population was achieved, with a kill rate exceeding 99.9%. These results demonstrated that FeLac is not only effective against *Cutibacterium acnes*, but also exhibits activity against other skin-associated microorganisms, indicating a broad antimicrobial spectrum. This comprehensive antimicrobial profile is particularly advantageous for preventing secondary infections and managing the complex microbial environment associated with compromised skin barriers.


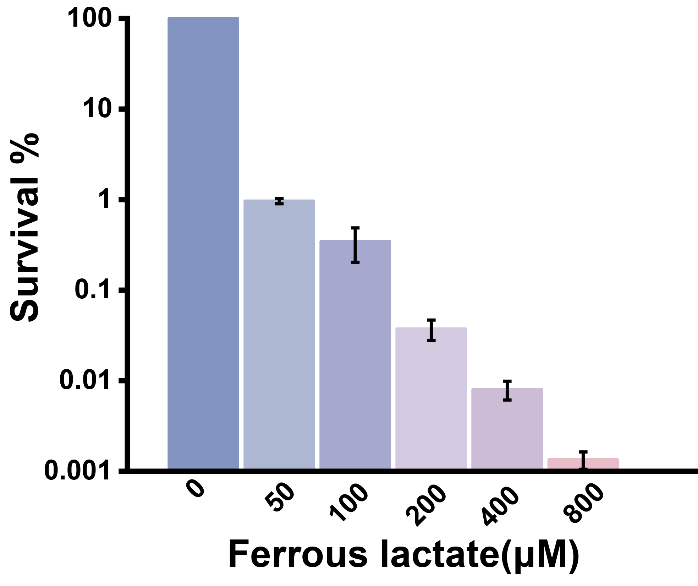


Figure S1. Survival of *Staphylococcus epidermidis* (RP62A) cells after exposure to FeLac at 200 μM for 3 h.
